# Alleviation of Shade Stress in Chinese Yew (*Taxus chinensis*) Seedlings with 5-Aminolevulinic Acid (ALA)

**DOI:** 10.3390/plants12122333

**Published:** 2023-06-15

**Authors:** Liuliu Wu, Linlin Song, Lifan Cao, Li Meng

**Affiliations:** 1 College of Life Science and Technology, Henan Institute of Science and Technology, Xinxiang 453003, China; 2 Engineering and Technology Research Center of Paper Mulberry Industry, Henan Academy of Sciences, Zhengzhou 451451, China

**Keywords:** yew, shade stress, antioxidase activities, reactive oxygen species, secondary metabolites

## Abstract

5-aminolevulinic acid (ALA) is a novel regulator that can promote plant growth, nitrogen uptake, and abiotic stress tolerance. Its underlying mechanisms, however, have not been fully investigated. In this study, the effects of ALA on morphology, photosynthesis, antioxidant systems, and secondary metabolites in two cultivars of 5-year-old Chinese yew (*Taxus chinensis*) seedlings, ‘Taihang’ and ‘Fujian’, were examined under shade stress (30% light for 30 days) using different doses of ALA (0, 30, and 60 mg/L). The findings from our study show that shade stress significantly reduced plant height, stem thickness, and crown width and increased malondialdehyde (MDA) levels. However, the application of 30 mg/L ALA effectively mitigated these effects, which further induced the activity of antioxidant enzymes under shade stress, resulting in the activities of superoxide dismutase (SOD), peroxidase (POD) and catalase (CAT) being increased by 10%, 16.4%, and 42.1%, and 19.8%, 20.1%, and 42% in ‘Taihang’ and ‘Fujian’, respectively. It also promoted their role in the absorption, conversion, and efficient use of light energy. Additionally, the use of 30 mg/L ALA caused a significant increase in the concentration of secondary metabolites, including polysaccharide (PC), carotenoid (CR), and flavonoids (FA), with increases of up to 46.1%, 13.4%, and 35.6% and 33.5%, 7.5%, and 57.5% in both yew cultivars, respectively, contributing to nutrient uptake. With ALA treatment, the yew seedlings showed higher chlorophyll (total chlorophyll, chlorophyll a and b) levels and photosynthesis rates than the seedlings that received the shade treatment alone. To conclude, the application of 30 mg/L ALA alleviated shade stress in yew seedlings by maintaining redox balance, protecting the photorespiratory system, and increasing organic metabolites, thus increasing the number of new branches and shoots and significantly promoting the growth of the seedlings. Spraying with ALA may be a sustainable strategy to improve the shade-resistant defense system of yew. As these findings increase our understanding of this shade stress response, they may have considerable implications for the domestication and cultivation of yew.

## 1. Introduction

Chinese yew (*Taxus chinensis*) is a type of dioecious gymnosperm that can tolerate environmental stresses and adapt to various environmental conditions [1]. Despite their extremely high medicinal value [2], some yew species are now extinct due to their low regeneration rates [3], slow seedling growth, and the over-exploitation and destruction of native habitats [4,5]. In its native habitat, yew usually occurs as a dispersed understory species [6]. Adequate adaptation to changing environmental conditions is a prerequisite for yew survival and competition in the wild. Observations on the development and survival status of yew seedlings in low-light conditions suggest that the lack of light observed in many areas is a major factor impeding seedling regeneration [7]. However, it is widely accepted that light restriction in shade-tolerant yew is ambiguous [8].

Plants under low-light conditions exhibit external morphologies such as fading leaf color, thinning leaves, slow growth, reduced resistance, and susceptibility to disease [9]. Previous studies have shown that low light can damage a series of photosynthesis apparatuses in plants, including the thylakoid membrane and the photosynthetic reaction center. Under weak or intense light saturation and compensation points, plants’ capability for non-photochemical bursts and electron transfer decreases. In addition, the net photosynthesis rate (Pn) decreases with an increase in photosynthetic rate, where plants exhibit reduced photorespiration and a decreased transpiration rate (Tr), as well as hydrocarbon accumulation and other physiological changes [10,11].

Shade-induced reactive oxygen species (ROS) oxidize the plant cytosol and increase cell membrane permeability. In response to poor light conditions, plants have adapted a variety of pathways to regulate their growth, physiology, and metabolism [12,13]. One of the most effective ways to reduce oxidative damage under shade stress is to enhance the antioxidant defense system, thereby reducing the prevalence of ROS. Previous studies have reported that lavender (*Kaempferia parviflora*) and vanilla (*Vanilla planifolia*) in shade-tolerant regions have greater antioxidant enzyme activities, including those of superoxide dismutase (SOD), peroxidase (POD), and catalase (CAT) [14,15]. In addition to antioxidant enzymes, some organic metabolites with strong antioxidant properties, such as proanthocyanidins (PCs), carotenoids (CRs), and flavonoids (FAs), play a vital part in scavenging ROS and reducing oxidative damage in plant cells [12]. Additionally, PA is sensitive to environmental conditions such as light, heat, and pH and plays an essential function in plant resistance to abiotic stresses [16]. CRs can absorb and dissipate excess light energy to protect the photosynthetic mechanism from high light damage [17]. CRs are the most effective single linear oxygen-bursting agents [18]. Studies have shown that FAs have great potential to inhibit ROS production and exhibit a higher ROS scavenging rate compared to vitamin C and E analogues [19]. Cultivation in shady places exerts environmental stress on vegetation and changes the balance between chlorophyll a and chlorophyll b concentrations [20]. Compared to plants grown under normal conditions, by improving the chlorophyll concentrations per unit surface area, the levels of chlorophyll a and b in plants can be increased to enhance their capacity to trap photons, thus helping plants make better use of light [21].

5-aminolevulinic acid (ALA) possesses similar physiological activity to plant hormones [22]. For instance, it can regulate chlorophyll synthesis, enhance the stability of chlorophyll and the PSⅡ consumption system, improve photosynthesis efficiency, promote photosynthesis and plant tissue differentiation, inhibit photorespiration in the dark, and enlarge pores, in addition to other basic physiological activities [23]. Therefore, exogenous ALA can be supplemented when adverse factors are present to promote chlorophyll synthesis and transformation and increase photosynthesis in plants.

As far as we know, there are no studies specifically showing the effects of ALA on shade-related stress tolerance in yew. In addition, the mechanisms of ALA that promote stress tolerance in plants are yet to be fully elucidated. To shed more light on these issues, this study aimed to compare two light application methods and determine the optimum ALA concentration that would provide the best protection against shade stress. Thus, we investigated the effects of ALA on the morphology, photosynthesis, antioxidant systems, and secondary metabolites of yew under shade stress. This study provides the first evidence that ALA can protect yew against shade stress, increasing our understanding of the role of ALA in promoting shade tolerance.

## 2. Results

### 2.1. Changes in Visual Quality Index in Response to Shade Stress

Our experiments demonstrated that shade stress can significantly change the visual growth quality of yew (Table 1). Under normal conditions, the two cultivars grew extremely slowly, and the rates of leaf necrosis and leaf litter were reported to be 10–15%. In addition, the number of new shoots and branches was significantly lower than that of the yew cultivars growing under shade stress. Leaf necrosis and leaf loss rates in the two yew cultivars treated with 0, 30, and 60 mg/L ALA were lower than 5% under shade stress. The number of new shoots and branches was highest in the two yew cultivars treated with 30 mg/L ALA. The two cultivars treated with ALA grew rapidly, and leaf necrosis and leaf litter rates were less than 5%. Thus, it was concluded that the concentration of ALA had a curvilinear correlation with visual growth quality.

### 2.2. Changes in Growth Parameters in Response to Shade Stress

**Figure 1:**
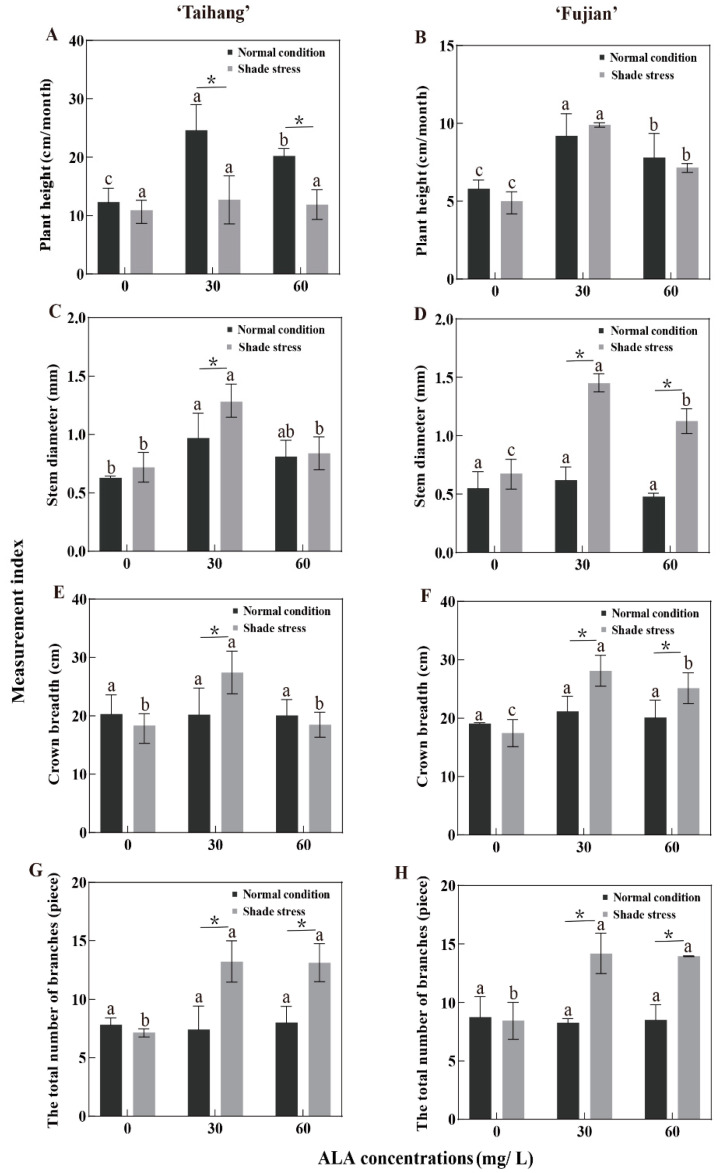
ALA’s effects on (**A**,**B**) plant height, (**C**,**D**) stem diameter, (**E**,**F**) crown breadth, and (**G**,**H**) the total number of branches of five-year-old Chinese yew cultivars after 30 days under shade stress. The vertical bar denotes the mean ± SE (*n* = 12). The letters (a–c) indicate that the three concentration gradients of ALA were significantly different (*p* ≤ 0.05) under normal conditions or shade stress. An asterisk (*) above the letter indicates a significant difference (*p* ≤ 0.05) in one particular cultivar between normal conditions and shade stress.

Under shade stress, there was no significant effect of ALA spraying on the pH of the ‘Taihang’ cultivar (Figure 1A); however, the pH of the ‘Fujian’ cultivar sprayed with 30 mg/L ALA was significantly higher than at other concentrations (Figure 1B). The SD, CB, and TNB of the ‘Taihang’ and ‘Fujian’ cultivars were significantly increased after spraying with 30 mg/L ALA under normal conditions (Figure 1C–H). Shade stress decreased the pH of the ‘Taihang’ and ‘Fujian’ cultivars by 18.2% and 15.1%, respectively, in comparison to normal light (Figure 1A,B). The SD of both cultivars increased from normal conditions to 14.3% and 25.5% under shade stress (Figure 1C,D). However, the CB and TNB were reduced by 6% and 4.8% and 4.4% and 17.6%, respectively (Figure 1E–H). Compared to normal conditions, after spraying with 30 mg/L ALA under shade stress, the SD, CB, and TNB of the ‘Taihang’ and ‘Fujian’ cultivars were significantly increased by 31.9%, 24.5%, and 91.7%, and 133.9%, 31.3%, and 77.5%, respectively (Figure 1C–H). Similarly, after spraying with 60 mg/L ALA, the SD, CB, and TNB of the ‘Fujian’ cultivar increased significantly under shade conditions compared with normal conditions, reaching 135.4%, 36%, and 78.5%, respectively (Figure 1D,F,H).

### 2.3. Changes in Gas Exchange and Photosynthetic Parameters in Response to Shade Stress

**Figure 2:**
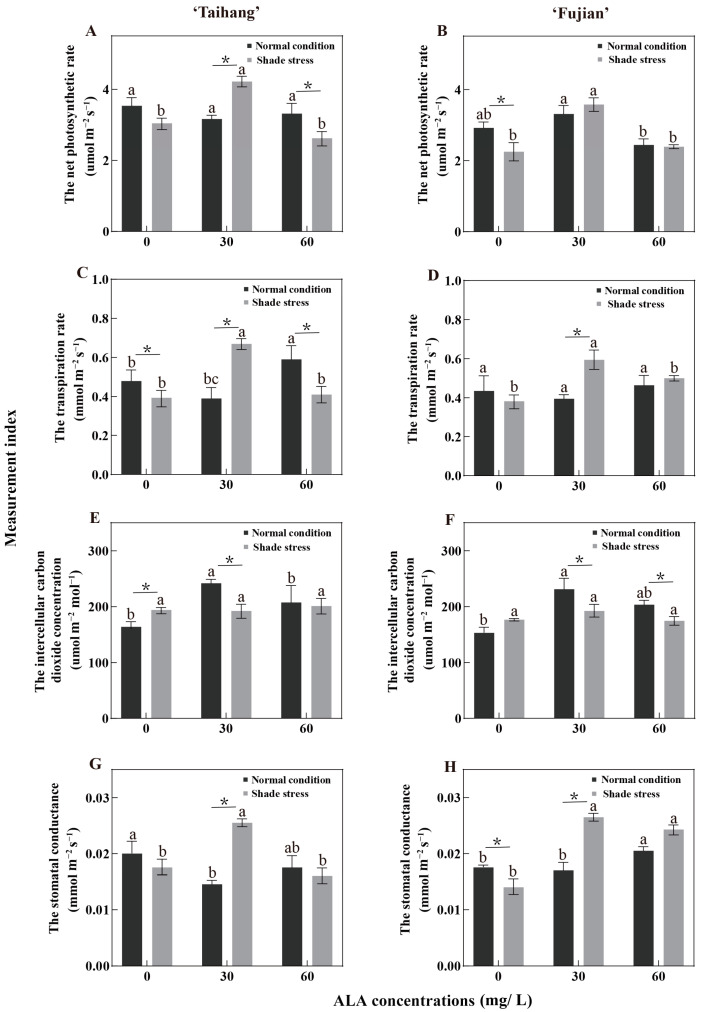
ALA effects on (**A**,**B**) the net photosynthetic rate, (**C**,**D**) the transpiration rate, (**E**,**F**) the intercellular carbon dioxide concentration, and (**G**,**H**) the stomatal conductance of five-year-old Chinese yew cultivars after 30 days under shade stress. The vertical bar denotes the mean ± SE (*n* = 12). The letters (a–c) indicate that the three concentration gradients of ALA were significantly different (*p* ≤ 0.05) under normal conditions or shade stress. An asterisk (*) above the letter indicates a significant difference (*p* ≤ 0.05) in one particular cultivar between normal conditions and shade stress.

Under shade conditions, the Pn, Tr, and Gs of the two yew cultivars were increased after spraying with 30 mg/L ALA (Figure 2A–D,G,H). The Ci in the ‘Taihang’ and ‘Fujian’ cultivars demonstrated no significant change under shade stress (Figure 2E,F). Shade stress reduced the Pn, Tr, and Gs in the two cultivars by 8.5%, 20.8%, and 10% and by 16.5%, 15.2%, and 11.8%, respectively, when compared with normal conditions (Figure 2A–D,G,H). The Ci of the ‘Taihang’ and ‘Fujian’ cultivars was slightly higher under shade stress than normal light, increasing by 13.9% and 24.7%, respectively (Figure 2E,F). The Pn of the yew cultivars under shade stress was significantly higher than that under normal conditions, increasing by 55.7% and 12.1% when sprayed with 30 mg/L ALA (Figure 2A, B). However, when compared to normal conditions, the Ci of the ‘Taihang’ and ‘Fujian’ cultivars decreased by 21.8% and 18.7% and 22.2% and 11.3%, respectively, under shade stress after spraying with 30 or 60 mg/L ALA (Figure 2E,F).

**Figure 3:**
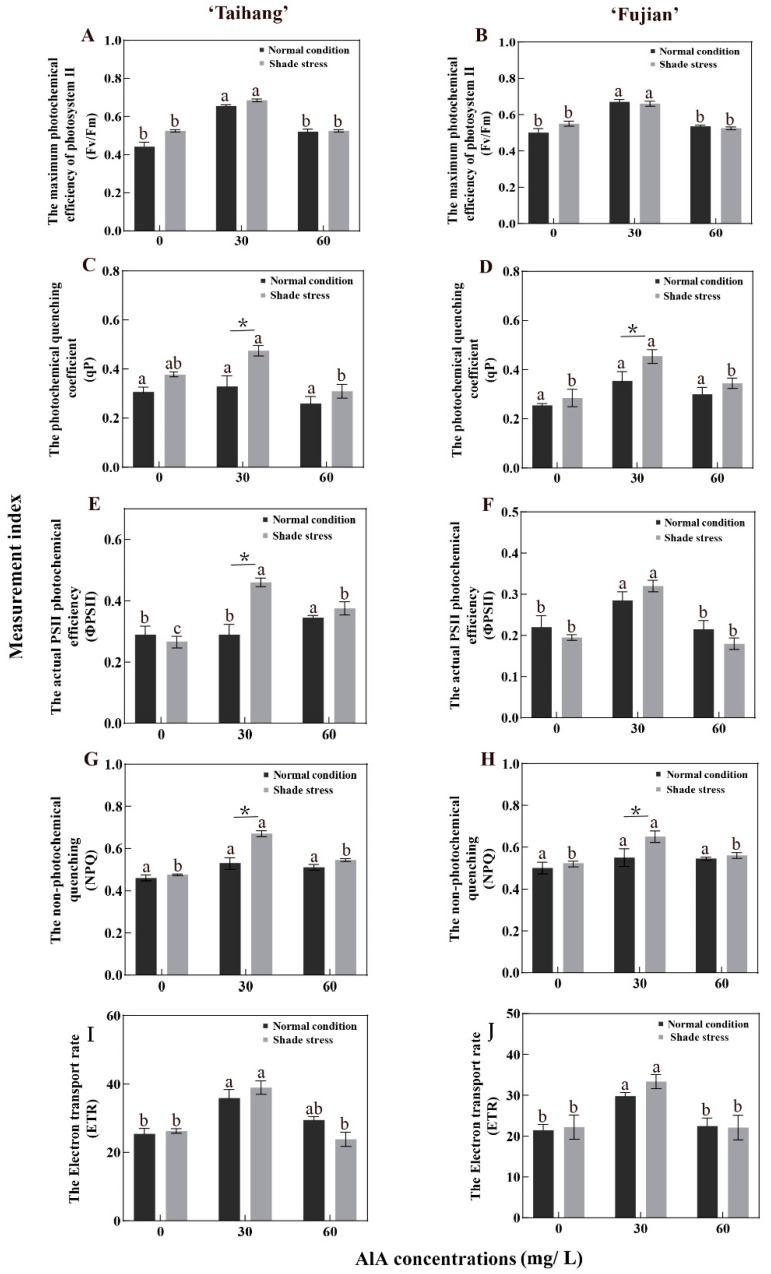
ALA effects on (**A**,**B**) the maximum photochemical efficiency of photosystem II, (**C**,**D**) the photochemical quenching coefficient, (**E**,**F**) the actual PSII photochemical efficiency, (**G**,**H**) the non-photochemical quenching, and (**I**,**J**) the electron transport rate of five-years-old Chinese yew cultivars after 30 days under shade stress. The vertical bar denotes the mean ± SE (*n* = 12). The letters (a–c) indicate that the three concentration gradients of ALA were significantly different (*p* ≤ 0.05) under normal conditions or shade stress. An asterisk (*) above the letter indicates a significant difference (*p* ≤ 0.05) in one particular cultivar between normal conditions and shade stress.

Under shade stress, the Fv/Fm, qP, ΦPSII, NPQ, and ETR of the two cultivars were increased compared to other concentrations when sprayed with 30 mg/L ALA (Figure 3A–J). The qP and NPQ in the ‘Taihang’ and ‘Fujian’ cultivars demonstrated no significant change under normal light (Figure 3G,H). However, after spraying with 30 mg/L ALA, the ETR of the ‘Taihang’ and ‘Fujian’ cultivars was significantly higher than with other treatments under normal conditions (Figure 3I,J). Shade stress increased Fv/Fm by 24.4% and 10% in the ‘Taihang’ and ‘Fujian’ cultivars, respectively, compared to normal light conditions (Figure 3A,B). The NPQ of the yew cultivars was slightly lower under shade stress than under normal conditions (Figure 3G,H). Shade stress was associated with a higher qP, ΦPSII, and ETR than normal light in both cultivars (Figure 3C–F,I,J). The Fv/Fm of the ‘Taihang’ cultivar after spraying with 30 mg/L ALA was also slightly higher than that of normal conditions, increasing by 4.5% (Figure 3A). Shade stress increased the qP, ΦPSII, and ETR of the ‘Taihang’ and ‘Fujian’ cultivars by 43.9%, 39.39%, and 8.5%, and 28.2%, 10.34%, and 11.8%, respectively, compared to normal conditions when sprayed with 30 mg/L ALA (Figure 3C–F,I,J), while the NPQ was decreased by 13.43% and 9.5%, respectively (Figure 3E,F). It also was observed that after spraying with 60 mg/L ALA, shade stress caused the NPQ of the ‘Taihang’ and ‘Fujian’ cultivars to be reduced by 1.75% and 1.72%, respectively, compared to normal light conditions (Figure 3F).

### 2.4. Changes in Antioxidant Enzyme Activities and MDA Content in Response to Shade Stress

**Figure 4:**
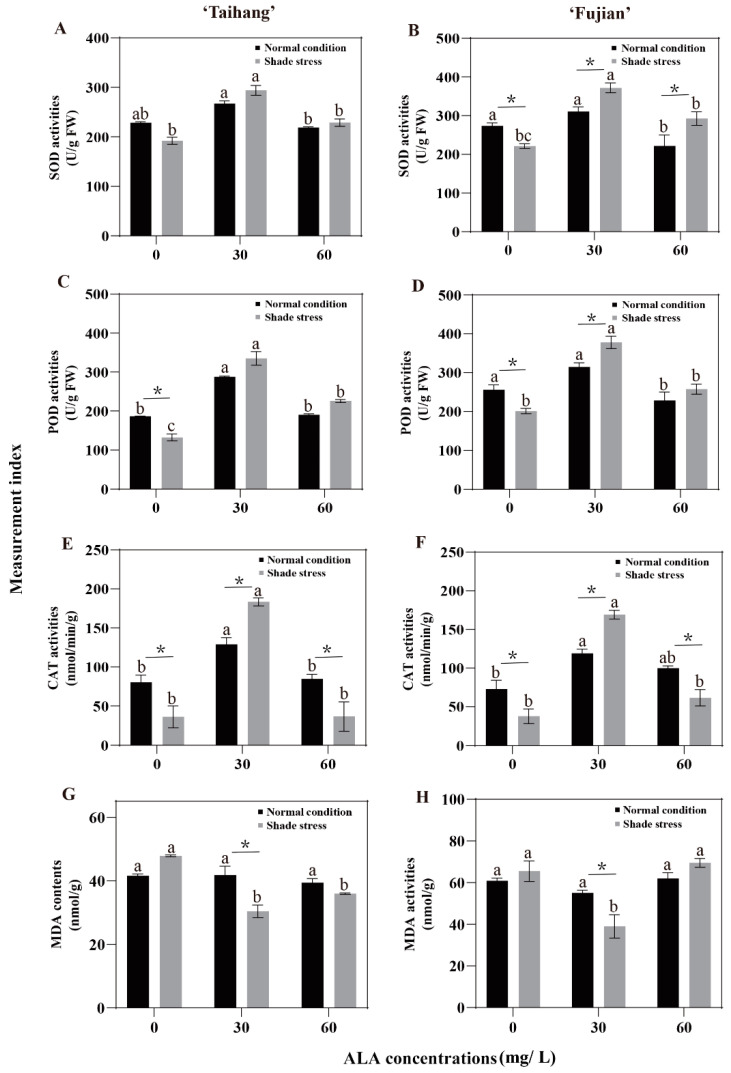
ALA effects on (**A**,**B**) SOD (superoxide dismutase) activities, (**C**,**D**) POD (peroxidase) activities, (**E**,**F**) CAT (catalase) activities, and MDA contents (**G**,**H**) of five-year-old Chinese yew cultivars after 30 days under shade stress. The vertical bar denotes the mean ± SE (*n* = 12). The letters (a–c) indicate that the three concentration gradients of ALA were significantly different (*p* ≤ 0.05) under normal conditions or shade stress. An asterisk (*) above the letter indicates a significant difference (*p* ≤ 0.05) in one particular cultivar between normal conditions and shade stress.

The SOD, POD, and CAT activities in the ‘Taihang’ and ‘Fujian’ cultivars treated with 30 mg/L ALA were significantly higher than those of yews treated with 0 and 60 mg/L ALA under normal conditions or shade stress (Figure 4A–F). However, the MDA content showed a significant decrease compared to the other concentration treatments (Figure 4G,H). The SOD, POD, and CAT activities under shade stress were 16.3%, 29.1%, and 54.9% and 18.9%, 21.3%, and 48.4% lower than normal conditions in the ‘Taihang’ and ‘Fujian’ cultivars, respectively. Meanwhile, shade stress increased MDA content by 26.2% and 8.3%, respectively. Applying a 30 mg/L ALA spray to both yew cultivars significantly increased SOD, POD, and CAT activities by 10%, 16.4%, and 42.1% and by 19.8%, 20.1%, and 42%, respectively (Figure 4A–F). The same trend was also observed for SOD and POD activities in the ‘Taihang’ and ‘Fujian’ cultivars when sprayed with 60 mg/L ALA under shade stress (Figure 4A–D). Nevertheless, spraying with 60 mg/L ALA decreased the CAT of both cultivars by 56.7% and 38.2%, respectively, under shade stress compared to normal conditions (Figure 4E,F). Additionally, MDA content was strongly stimulated after spraying with 30 mg/L ALA, while MDA was reduced by 27.2% and 29.1% in both cultivars under shade stress compared to normal conditions (Figure 4G,H).

### 2.5. Changes in Chlorophyll Concentration in Response to Shade Stress

**Figure 5:**
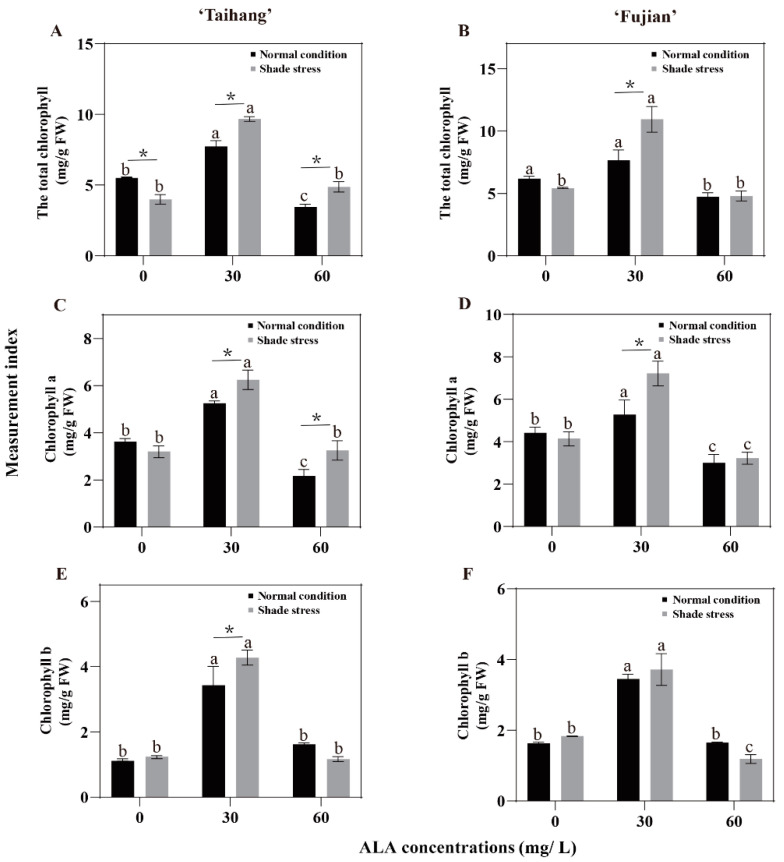
ALA effects on (**A**,**B**) the total chlorophyll concentration, (**C**,**D**) the chlorophyll a concentration, and (**E**,**F**) the chlorophyll b concentration of five-year-old Chinese yew cultivars after 30 days under shade stress. The vertical bar denotes the mean ± SE (*n* = 12). The letters (a–c) indicate that the three concentration gradients of ALA were significantly different (*p* ≤ 0.05) under normal conditions or shade stress. An asterisk (*) above the letter indicates a significant difference (*p* ≤ 0.05) in one particular cultivar between normal conditions and shade stress.

Under normal conditions and shade stress, the concentrations of total Chl, Chl-a, and Chl-b were significantly higher in both cultivars treated with 30 mg/L ALA than in yews exposed to 0 and 60 mg/L ALA (Figure 5A–F). Shade stress reduced the total Chl in the ‘Taihang’ and ‘Fujian’ cultivars by 29.1% and 11.8%, respectively, compared with normal light (Figure 5A,B). In the ‘Taihang’ and ‘Fujian’ cultivars, the Chl-a concentration was slightly lower under shade stress than it was under normal conditions. Shade stress increased Chl-b by 12.7% in the ‘Taihang’ cultivar and 22.7% in the ‘Fujian’ cultivar, compared to normal light. Spraying with 30 mg/L ALA significantly increased the total Chl of both yew cultivars by 25.2% and 25.3% (Figure 5A,B). Simultaneously, the Chl-a concentration was significantly increased under shade stress by 19% and 36.7% (Figure 5C,D). Regarding the use of 30 mg/L ALA, shade stress increased the Chl-b concentration of the two cultivars compared to under normal conditions (Figure 5E,F). However, spraying with 60 mg/L reduced Chl-b by 27.8% and 28% in the ‘Taihang’ and ‘Fujian’ cultivars, respectively (Figure 5E,F).

### 2.6. Changes in Secondary Metabolites in Response to Shade Stress

**Figure 6:**
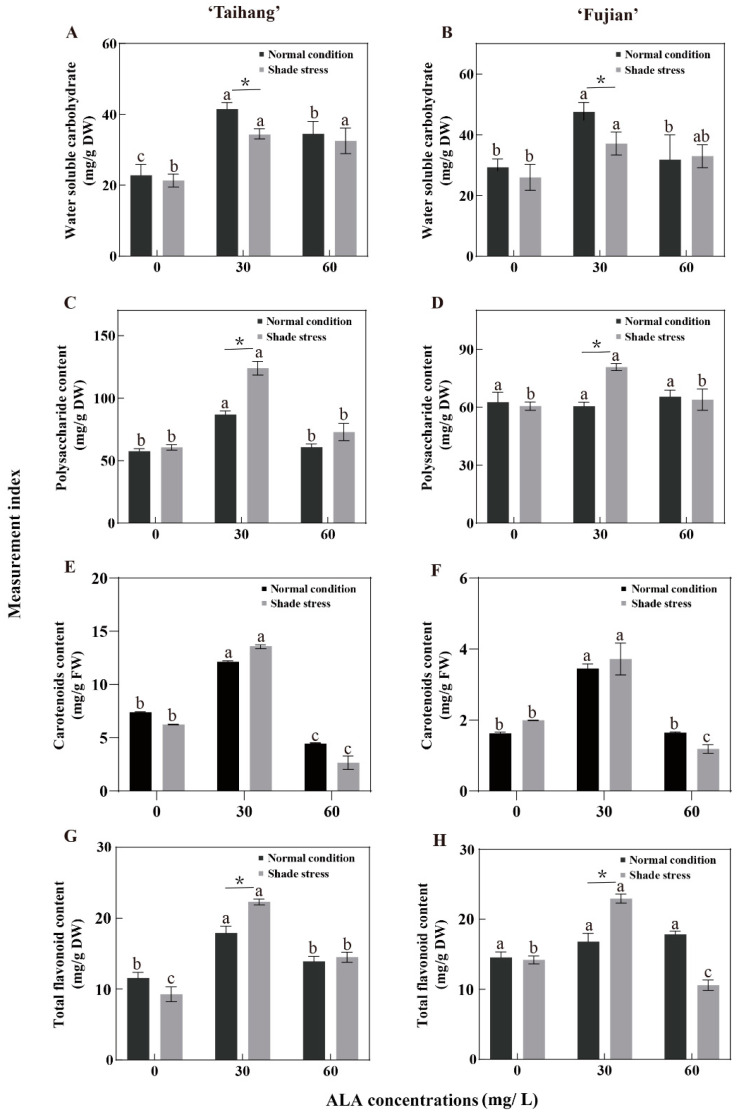
ALA effects on (**A**,**B**) the water-soluble carbohydrate concentration; (**C**,**D**) the polysaccharide concentration; (**E**,**F**) the carotenoid concentration; and (**G**,**H**) the flavonoid concentration of five-year-old Chinese yew cultivars after 30 days under shade stress. The vertical bar denotes the mean ± SE (*n* = 12). The letters (a–c) indicate that the three concentration gradients of ALA were significantly different (*p* ≤ 0.05) under normal conditions or shade stress. An asterisk (*) above the letter indicates a significant difference (*p* ≤ 0.05) in one particular cultivar between normal conditions and shade stress.

Under normal conditions and shade stress, the concentrations of WSC and CR were significantly higher in both cultivars treated with 30 mg/L ALA than in yews treated with 0 and 60 mg/L ALA (Figure 6A,B,E,F). Under natural conditions, the different concentrations of ALA had almost no effect on the PC concentration in the ‘Fujian’ cultivar; however, the PC concentration in the ‘Taihang’ cultivar sprayed with 30 mg/L ALA was significantly higher than those treated with the other concentration treatments (Figure 6C,D). The same phenomenon occurred with the determination of FA concentrations (Figure 6G,H). Shade stress did not cause significant changes in the WSC concentration in the ‘Taihang’ cultivar compared to normal conditions, while WSC increased by 14.2% in the ‘Fujian’ cultivar (Figure 6A,B). In terms of PC concentration, no significant change was observed between shade stress and normal light in both the ‘Taihang’ and ‘Fujian’ cultivars (Figure 6C,D). The CR concentration in the ‘Taihang’ cultivar under normal conditions was lower than that under shade stress, decreasing by 15.7%, while it was increased by 22.8% in the ‘Fujian’ cultivar (Figure 6E,F). In addition, the FA concentration in the ‘Taihang’ cultivar was decreased by 19.9% under normal conditions. Under shade stress, the concentrations of WSC and PC were significantly higher than under normal conditions in both the ‘Taihang’ and ‘Fujian’ cultivars, increasing by 20.3% and 46.1% and by 52.9% and 33.5%, respectively, after spraying with 30 mg/L ALA (Figure 6A,B). The CR concentration in both cultivars was also significantly increased by 13.4% and 7.5% upon spraying with 30 mg/L ALA (Figure 6E,F). Under shade stress, the CR concentration was reduced by 52.5% in the ‘Taihang’ cultivar and 27.9% in the ‘Fujian’ cultivar after spraying with 60 mg/L ALA (Figure 6E,F). When 30 mg/L ALA was sprayed, the FA concentration in the ‘Taihang’ and ‘Fujian’ cultivars increased under normal conditions by 35.6% and 57.5%, respectively, compared with shade stress, whereas spraying with 60 mg/L ALA reduced the FA concentration in the ‘Fujian’ cultivar by 31.2%.

### 2.7. Stress Index, Hierarchical Clustering, and Principal Component Analysis in Response to Shade Stress

As shown in Table 2, the SI of the pH of the ‘Taihang’ cultivar was significantly lower than that of the control, with the ‘Fujian’ cultivar demonstrating opposing results. The SI of the SD and TNB in both cultivars was noticeably higher when ALA was applied at 30 mg/L. The SI of CB was not significantly affected by ALA under both types of light. With the application of 30 mg/L ALA, the SI of Pn, Tr, and Gs was significantly greater in both the ‘Taihang’ and ‘Fujian’ cultivars than under other treatments, while the SI of Ci was considerably lower. The SI of Fv/Fm, ΦPSⅡ, and qP was significantly increased under normal light, contrasting ALA application. The SI of the NPQ in both cultivars was decreased after spraying with ALA, and the SI of the ETR in the ‘Taihang’ cultivar was significantly higher than in the control after the application of 30 mg/L ALA. After the application of ALA, the SI of SOD, POD, and CAT was higher in the ‘Taihang’ cultivar than in the control. Comparatively, the SI of MDA was dramatically lowered. The total Chl and Chl-a in the ‘Taihang’ and ‘Fujian’ cultivars showed a higher SI with ALA treatment. In the ‘Taihang’ cultivar, with the addition of 30 mg/L ALA, the SI of WSC, PC, CR, and FA was higher than under the other treatments, while in the ‘Fujian’ cultivar, the spraying of ALA reduced the SI of CR.

**Figure 7:**
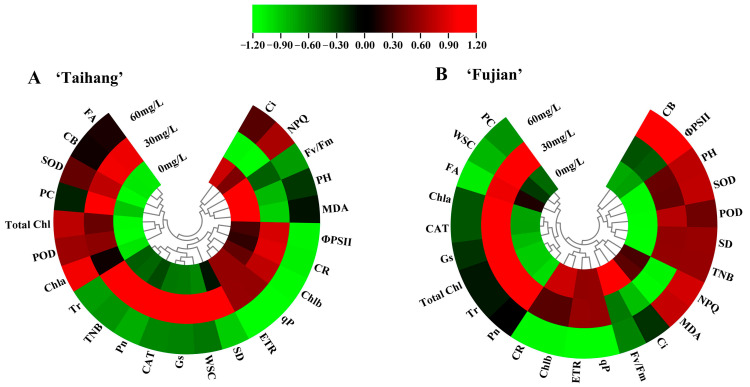
Heatmap and hierarchical clustering of physiologic and morphologic parameters in three concentration gradients of ALA of five-year-old (**A**) ‘Taihang’ and (**B**) ‘Fujian’ cultivars after 30 days under shade stress. The color gradient indicates the degree of response to ALA level (from low to high).

**Figure 8:**
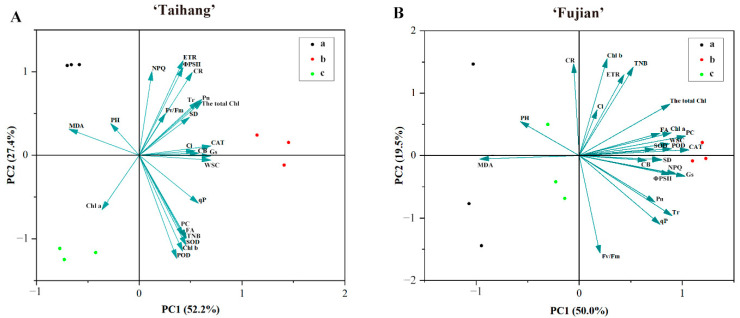
Principal component analysis of the stress index (SI) in three concentration (a,b,c; 0,30,60 mg/L) gradients of ALA of five-year-old (**A**) ‘Taihang’ and (**B**) ‘Fujian’ cultivars after 30 days under shade stress.

The heat maps in Figure 7A,B display a hierarchical clustering analysis of 24 parameters under shade stress. In both cultivars, the variation curves of the control group and the 60 mg/L ALA group were more similar than those of the 30 mg/L ALA group (Figure 7A). The addition of 30 mg/L ALA under shade stress resulted in a similar variation profile in both cultivars (Figure 7A,B). Under shade stress, for PCA, in the ‘Taihang’ cultivar, PC1 and PC2 were found to explain 52.2% and 27.4% of the overall variation, respectively (Figure 8A). The POD, SOD, Chl-a, Chl-b, total Chl, CR, ETR, Pn, Gs, and TNB varied more than other parameters (Figure 8A). PC1 and PC2 for the ‘Fujian’ cultivar accounted for 50% and 19.5% of the overall variation, respectively (Figure 8B). In reaction to shade stress, the MDA, CAT, TNB, Chl-b, Fv/Fm, PC, CR, Gs, Tr, and total Chl were less variable than any of the other measures (Figure 8B).

## 3. Discussion

Weak light causes several physiological and biochemical metabolic processes to be disrupted in plants, resulting in leaf curling, a loss of greenery, and a decrease in plant production and biomass [24,25]. Previous studies have demonstrated that shade stress damages yew seedlings (Table 1). Meanwhile, ALA’s effects on plants at the physiological, biochemical, and molecular levels led to its discovery as a new plant growth regulator [26]. ALA has been demonstrated to successfully protect plants from the negative impacts of abiotic stressors [27]. The purpose of this study was to investigate how ALA protects yew seedlings from shade stress. We found that natural light and shade stress reduced the concentrations of growth indicators in yew (Figure 1). ALA effectively alleviated shade stress, particularly at a concentration of 30 mg/L, by increasing the number of branches, stem thickness, and crown breadth and shortening the main stem length (Figure 1). It has been noted that bermudagrasses under shade stress produce more biomass when exogenous ALA is applied [12]. These findings support the protective function of ALA in promoting yew tolerance to shade stress.

In addition to changes in morphology, changes in leaf chlorophyll concentration and photosynthesis factors are necessary to maintain normal growth and photosynthesis and are important strategies for shade resistance in plants [28,29]. Although lower levels of light intensity can be effective in promoting the formation of Chl in the short term, long periods of low light inhibit the biosynthesis of Chl and accelerate its degradation, leading to leaf shrinkage [30,31]. After ALA treatment, we observed a remarkable rise in total Chl concentration, as well as Chl-a and Chl-b concentration, in yew seedlings under shade stress (Figure 5). Thus, the application of ALA helped the yew to capture and utilize more light energy, giving the plant an advantage in shady conditions, representing one important adaptive mechanism for yew seedlings in shady conditions.

Photosynthetic capacity is strongly associated with the maintenance of vegetative growth under stress, as the photosynthetic process provides the plant with an energy supply through the assimilation of carbon dioxide [32]. In response to a situation of environmental stress, plants adapt the closing and opening of their stomata to maintain a steady state of water and gas exchange [32]. The Pn and Tr showed a tendency to be consistent with Gs, as they are essential indicators of water vapor and gas exchange [33]. In the present study, shade stress reduced the Pn, Gs, and Tr in yew seedlings and increased Ci (Figure 2). The application of 30 mg/L ALA resulted in a significant increase in Pn, Gs, and Tr and a decrease in Ci. Chlorophyll fluorescence parameters, such as Fv/Fm, qP, ΦPSII, NPQ, and ETR, are a set of variables or constants used to describe plants’ photosynthetic mechanisms and photosynthetic physiology, reflecting the internal characteristics of the plants. They can also reflect the health of chloroplasts under conditions of stress, which include heat, drought, salt, and shade [34]. In this study, shading reduced the NPQ of yew seedlings (Figure 3). Additionally, we also found that treatment with ALA (30 mg/L) increased Fv/Fm, qP, ΦPSII, and ETR in yew seedlings under shade stress situations (Figure 3, Figure 7, and Figure 8). Because the reduction in fluorescence parameters affected the reproduction and degeneration of photosynthesis organs, it reduced the efficiency of light conversion and thus inhibited photosynthesis. ALA promotes donor-side PSII reaction center activity and PSII electron transfer itself, increasing the intensity of photosynthesis [35]. Our findings were also comparable to previous results regarding the impact of extreme temperatures [35,36]. These findings suggest that exogenous ALA can help maintain high light energy utilization, promote photosynthesis, and ultimately increase the biomass of yew seedlings and improve the adaptation of yew to shade stress.

ALA was previously reported to reduce oxidative stress damage caused by ROS accumulation in shaded environments [37]. MDA is the major product of peroxidation in the membrane system and is a key indicator of lipid peroxidation in membranes. In the present investigation, we found that shade stress increased the MDA content of yew seedlings compared to natural conditions. The exogenous application of 30 mg/L ALA significantly reduced their MDA content (Figure 4G,H, Figure 7 and Figure 8). It has been shown that ALA can reduce the MDA levels in kale-type oilseed rape by increasing antioxidant enzyme concentrations under shade stress [35,38,39,40]. These results suggest that ALA can enhance the shade tolerance of yew seedlings by reducing redox damage. Plants are equipped with enzymatic and non-enzymatic systems that decrease damage to their cells by reacting to ROS in the cells when subjected to stress [41]. Shade-induced oxidative stress can trigger systems involving a variety of antioxidant enzymes and non-enzymatic activators. In the present study, it was shown that shade stress reduced enzymatic antioxidant (SOD, POD, and CAT) activities in yew seedlings (Figure 4). It has been reported that the foliar application of ALA in sunflower hybrids can decrease yield loss due to drought and increase oil concentrations by increasing the activities of SOD, POD, and CAT [42,43]. Thus, ALA is essential for regulating plants’ antioxidant capacity and redox homeostasis under adverse conditions. These results suggest that spraying with ALA (30 mg/L) can increase the tolerability of yew seedlings to shade stress by improving non-enzymatic oxidant levels and the activities of antioxidant enzymes.

WSC is a product of photosynthesis. Precipitation, growing temperature, and the duration and intensity of sunlight exposure affect WSC concentration [44]. The present study demonstrated that natural light or shade resulted in a reduction in WSC due to higher energy consumption in the yew under stress, while spraying with ALA (30 mg/L) improved the shade tolerance of yew seedlings. CRs are lipid-soluble pigments that, to a certain degree, define the color of an organism. The primary function of CRs in the photosynthetic organs of higher plants is to convey light energy and extinguish excessive light energy, particularly under highly exposed light conditions [18]. CRs have also been shown to be strong antioxidants, killing singlet oxygen and protecting photosynthetic membranes from redox damage [28]. PC is one of the many FA components of plants. Both PC and FA have very powerful antioxidant capacities, scavenging superoxide anions and free hydroxyl radicals and protecting against ROS attacks on lipids [45]. Previous studies have shown that FA and PC increase oxidative activity and scavenge hyperoxide anion radicals and superoxide radicals to mitigate shade-induced redox damage [46,47]. Here, compared with natural conditions, shade stress reduced the CR concentration in yew seedlings (Figure 6), whereas the application of 30 mg/L ALA significantly increased the CR concentration in yew, which may be because spraying with ALA enables yews to have a greater antioxidant and photoprotective capacity in response to shade stress. This was also shown using hierarchical clustering and principal component analysis (Figure 7 and Figure 8). Additionally, the application of ALA maintained a significant increase in PC and FA concentrations in yew seedlings and promoted their adaptation to shady environments (Figure 6).

## 4. Conclusions

Exogenous ALA application can improve shade stress tolerance in yew seedlings by eliminating excess ROS and maintaining higher photosynthetic performance and gas exchange. Previous studies have shown that shade stress severely inhibits photosynthesis, reduces antioxidative capacities, and disturbs nutritional homeostasis, leading to oxidative stress in yew. The application of 30 mg/L ALA was used to stimulate the shade stress response in yew by improving photoenergy conversion efficiencies, increasing the concentrations of Chl, and scavenging ROS as well as regulating the activities of antioxidant enzymes, thus maintaining a high redox balance. In addition, ALA increased the concentration of organic metabolites (WSC, FA, PC, etc.) and facilitated nutrient uptake, thus enhancing the shade resistance of yew and promoting the growth of the seedlings. These differences in morphological and physiological status reflected the plants’ adaption to shade stress in the presence of the application of exogenous ALA. Deeper insights into the precise mechanism of ALA-mediated shade resistance in yew at the molecular level are needed.

## 5. Materials and Methods

### 5.1. Plant Material and Experimental Design

This study was conducted at the Institute of Traditional Chinese Medicine, Henan Institute of Science and Technology, Xinxiang, China (N 35°18′13.71, E 113°55′15.05). Yew cultivars, namely 5-year-old seedlings with consistent and healthy growth, were selected and transferred into pots. These basins were filled with 25 kg of fertile mixed soil collected from the topsoil to a depth of 30 cm from the yew’s native habitat. The soils used were of sandy and mixed types, containing 3.2% organic matter, 9.6% total nitrogen (N) concentration, 78 mg/kg available N, 23.1 mg/kg available phosphorus (P), and 143 mg/kg exchangeable potassium (K) concentration. The original domesticated plant was transferred to the experimental site after one year. The irrigation frequency was evaluated by considering the time in which the pots lost between 70% and 80% of the readily available water in the substrate, determined based on weight. Fertilization was conducted using irrigation heads, and nutrients were provided at constant concentrations in the irrigation water, which contained 20% CO(NH_2_)_2_, 3.89% NH_4_(NO_3_), 6.11% KNO_3_, and 10.00% CO(NH_2_)_2_.

Our experiment used two yew cultivars, ‘Taihang’ and ‘Fujian’, which are from the north and south of China, respectively. Artificial shade was used for the shade treatment (30% light), which was assessed along with a normal condition (natural light). Black shade netting (30%; Winton, China) was placed at nominal levels. The photosynthetically active radiation (PAR) values of the shade cloth were measured with LI-190 quantum sensors (LI-COR, Lincoln, NE, USA) and averaged to determine the actual PAR value of 35%. According to the study [48], 30 mg/L ALA can protect plants against oxidative damage under low-temperature stress. Therefore, when the yew seedlings had fully grown four new leaves, they were sprayed with 0, 30, and 60 mg/L concentrations of ALA solution until the leaves were completely wet on both sides and then placed under normal light. The same concentrations of ALA were applied under shade conditions. The indicators were measured after 30 days, where the spraying was repeated on the 15th day with the same concentration of ALA. Terminal shoots (including leaves and twigs) were all sampled under each of the different light conditions. The treatments were replicated three times, and six terminal shoots were collected from the top of each individual plant for analysis. Twelve individuals were sampled from each treatment for analysis, with all treatments using a randomized complete block design, in March 2022. Each treatment included five independent biological replications.

### 5.2. Determination of the Visual Quality Index

Visual indicators were used to evaluate aesthetic quality at the end of each stage. The index ranged from 1 (poor quality) to 5 (very good quality) based on the percentage of leaf discoloration, leaf necrosis, leaf litter, and new shoots and branches, according to Korkmaz et al. [49]. The cumulative leaf area collected during the experiment was recorded and calculated according to the leaf area percentage. The number of new shoots and branches was determined and recorded during the experiment for each plant. Then, the percentage of new shoots and branches was calculated for each plant. The grading of leaf discoloration was as follows: (1) >35%; (2) 25–35%; (3) 15–25%; (4) 5–15%; and (5) less than 5%. The grading of leaf necrosis and leaf litter was as follows: (1) >20%; (2) 15–20%; (3) 10–15%; (4) 5–10%; and (5) less than 5%. The average individual scores for leaf shedding, leaf discoloration, leaf necrosis, and the number of new shoots and branches were determined to obtain an overall visual quality score.

### 5.3. Determination of Plant Growth Indicators

Plant height (PH), stem diameter (SD), and crown breadth (CB) were measured using a ruler and Vernier calipers [48]. The total branch number (TNB) of fully expanded new branches was also calculated [50].

### 5.4. Determination of Gas Exchange Parameters and Chlorophyll Fluorescence Parameters

Leaf gas exchange and chlorophyll fluorescence parameters were measured using an LI-6400 XT Portable Photosynthesis System (LI-6400XT, LI-COR, Lincoln, NE, USA) according to White’s method [51]. The values of net photosynthetic rate (Pn), stomatal conductance (Gs), transpiration rate (Tr), and intercellular carbon dioxide concentration (Ci) were determined. During the measurements, a chamber attached to a leaf maintained in 380 mol CO_2_ mol^−1^ air, the leaf temperature was kept at 25 °C, the photosynthetic photon flux intensity was adjusted to 1000 μmol photons m^−2^ s^−1^, and the relative humidity reached 75%. After approximately 8 min, steady-state data were recorded. Before chlorophyll fluorescence was observed, the fully expanded leaves were acclimatized in the dark for 30 min. The maximal photochemical efficiency (Fv/Fm), photochemical quenching (qP), the actual photochemical efficiency of photosystem II (ΦPSII), non-photochemical quenching (NPQ), and electron transport rate (ETR) were computed [52]. The indicators were measured from 09:00 to 15:00 on the fourth fully expanded leaf of the terminal shoot from the top of each plant.

The chlorophyll (Chl) concentration was determined by taking fresh leaf samples (0.5 g) from three randomly selected leaves per replicate. As described by Du et al. [53], the samples were homogenized with 5 mL of acetone (80% *v*/*v*) using a pestle and mortar and filtered through a filter paper (Whatman No. 2). The absorbance was then measured with a UV/visible spectrophotometer (Spectramax Plus 384, Molecular Devices, San Jose, CA, USA) at 663 and 645 nm.

### 5.5. Determination of Antioxidant Systems and MDA Content

The antioxidant enzyme systems in the leaves of each fresh sample were evaluated by detecting the activities of superoxide dismutase (SOD, No. A001-3-2), peroxidase (POD, No. A084-3-1), catalase (CAT, No. A007-1-1), and malondialdehyde (MDA, No. A003-1-2) using test kits (Nanjing Jiancheng Bioengineering Institute, Nanjing, China) according to the manufacturer’s instructions.

### 5.6. Determination of Secondary Metabolites

According to Li et al. [54], the concentration of water-soluble carbohydrates (WSC) was measured. A fine powder was prepared from dried leaves; an aliquot of 0.5 g was added to 2 mL of 80% (*v*/*v*) ethanol, and the mixture was heated in a water bath for 40 min at 80 °C. The supernatant was removed via centrifugation at 5000× *g* for 10 min. The reaction mixture was heated to 100 °C in a water bath for 10 min using 1 mL of supernatant, 4 mL of 98% sulfuric acid, and 1 mL of 5% phenol. The concentration of WSC was determined at 490 nm using D-glucose as the reference.

Polysaccharide (PC) was determined based on the methodology of Bayar et al. [55]. Briefly, 0.5 g of powder was added to 25 mL of boiling water in a test tube and placed in an ultrasonic cleaning machine at 70 °C and 100 W for 40 min, awaiting extraction. The filtrate was fixed in a 100 mL volumetric flask. After diluting the solution by a factor of 25 to 50 mL in a constant volume bottle, 2 mL was added to a test tube and spectrophotometrically evaluated at 490 nm with phenol and concentrated sulfuric acid, as mentioned above.

The method for determining chlorophyll was used to assess the carotenoid (CR) level at 470 nm [53].

The aluminum chloride colorimetric method was used to determine the flavonoid (FA) content in yew extract [56]. In short, the samples were dried to a constant weight, then crushed and sieved. The yew extraction was replenished with 70 µL of 5% sodium nitrite solution, left to stand for 5 min, then combined with 1.3 mL of distilled water, 0.5 mL of 1M sodium hydroxide, and 0.15 mL of 10% aluminum chloride and held at room temp for 5 min. The OD was observed using a multimode plate reader at 510 nm (Synergy H1, BioTek, Winooski, VT, USA). Calibration curves were created using catechin as a reference standard.

### 5.7. Data Analysis

All data and principal component analysis (PCA) processes were conducted using the SPSS software (version 20.0; SPSS, Inc., Chicago, IL, USA). One-way analysis of variance (ANOVA) was performed on the data using Tukey’s method [57]. Significant differences between ALA concentrations of 0, 30, and 60 mg/L were evaluated. The letters (a–c) indicate that the three concentration gradients of ALA were significantly different (*p* ≤ 0.05) under normal conditions or shade stress. An asterisk (*) above the letter indicates a significant difference (*p* ≤ 0.05) in one particular cultivar between normal conditions and shade stress. The formula for the stress index (SI), which was used to measure the level of change in a certain parameter under stress vs. normal conditions, is SI = Stress parameter/Normal parameter x 100 [58]. Data from the heat map [12] were transformed using log_2_ (SI/100).

## Figures and Tables

**Table 1:** Effect of ALA applications via different methods on the visual growth state of five-year-old Chinese yew seedlings subjected to shade stress for 30 days.

Treatments	Visual Quality Index (1–5)
App. Method	
Normal conditions	2.68 ± 0.13 a
Shade stress	1.57 ± 0.21 c
Cultivar × 5-ALA concentrations	2.13 ± 0.15 b
‘Taihang’ (mg/L)	
0	0.55 ± 0.09 c
30	1.82 ± 0.21 a
60	1.14 ± 0.15 b
‘Fujian’ (mg/L)	
0	1.32 ± 0.13 c
30	2.98 ± 0.21 a
60	2.18 ± 0.15 b
ANOVA	
App. method (M)	**
ALA concentrations (C)	***
M × C	NS

Note: Different letters (a–c) indicate statistical differences between ALA concentrations at *p* ≤ 0.05 based on Tukey’s test. Asterisks (*** and **) indicate a significant difference at *p* ≤ 0.01 or 0.001 among three concentration gradients under normal conditions or shade stress, respectively; NS indicates no significant differences in the two cultivars between normal conditions and shade stress.

**Table 2:** Effect of ALA on the stress index (SI) of five-year-old Chinese yew cultivars after 30 days under shade stress.

Parameter	Normal Conditions						Shade Stress						Stress Index (%)					
	Taihang			Fujian			Taihang			Fujian			Taihang			Fujian		
	0	30	60	0	30	60	0	30	60	0	30	60	0	30	60	0	30	60
PH	12.48	24.67	20.20	5.82	9.24	7.83	10.21	12.72	11.96	4.94	9.93	8.84	82	52	59	85	107	113
SD	0.63	0.97	0.81	0.55	0.62	0.48	0.72	1.28	0.84	0.69	1.45	1.13	114	132	104	125	234	235
CB	20.31	20.22	20.10	19.22	27.42	18.49	19.09	21.18	20.11	18.38	28.13	25.14	94	105	100	96	103	136
TNB	8.30	7.43	8.03	8.76	8.28	7.90	7.90	14.24	7.04	7.22	14.70	14.10	95	192	88	82	178	178
Pn	3.87	2.71	3.32	2.61	3.31	2.44	3.54	4.22	2.63	2.18	3.71	2.35	91	156	79	84	112	96
Tr	0.48	0.39	0.59	0.46	0.40	0.47	0.38	0.67	0.41	0.39	0.58	0.49	79	172	69	85	145	104
Ci	173.45	245.54	207.53	148.58	251.44	203.56	197.51	192.00	201.33	185.34	195.57	180.50	114	78	97	125	78	89
Gs	0.02	0.02	0.02	0.02	0.02	0.02	0.02	0.03	0.02	0.02	0.03	0.02	90	173	89	88	159	105
Fv/Fm	0.41	0.66	0.52	0.50	0.67	0.54	0.51	0.69	0.53	0.55	0.66	0.53	124	105	102	110	99	98
qP	0.33	0.33	0.26	0.39	0.39	0.30	0.48	0.48	0.31	0.50	0.50	0.35	145	145	119	128	128	115
ΦPSII	0.23	0.33	0.35	0.19	0.29	0.21	0.30	0.46	0.38	0.22	0.32	0.18	130	139	109	116	110	86
NPQ	0.54	0.67	0.57	0.56	0.63	0.58	0.53	0.58	0.56	0.54	0.57	0.57	98	87	98	96	90	98
ETR	35.89	35.89	29.45	29.83	29.83	22.49	38.91	38.95	23.83	33.36	33.40	22.11	108	109	81	112	112	98
SOD	228.54	267.33	218.95	273.61	310.75	222.27	191.27	294.01	228.74	221.82	372.17	292.71	84	110	104	81	120	132
POD	186.78	288.09	190.75	255.92	314.76	228.67	132.44	335.37	226.02	201.33	378.09	257.78	71	116	118	79	120	113
CAT	80.46	129.04	84.75	73.33	119.11	99.95	36.32	183.33	36.69	37.86	169.30	61.72	45	142	43	52	142	62
MDA	41.96	41.76	39.42	64.54	55.00	62.12	52.94	30.39	35.96	65.57	39.00	69.54	126	73	91	102	71	112
Total Chl	5.35	7.73	3.44	6.18	8.73	4.74	3.79	9.68	4.87	5.45	10.94	4.80	71	125	142	88	125	101
Chla	3.57	5.25	2.17	3.86	5.28	3.19	3.18	6.25	3.25	3.65	7.22	3.22	89	119	150	95	137	101
Chlb	1.26	3.43	1.62	1.63	3.45	1.65	1.42	4.28	1.17	2.00	3.72	1.19	113	125	72	123	108	72
WSC	22.84	34.50	34.50	22.77	34.11	31.81	21.33	41.50	32.54	26.00	49.81	32.97	93	120	94	114	146	104
PC	57.59	80.99	60.75	63.59	60.47	65.41	60.50	123.89	72.89	60.50	80.75	59.89	105	153	120	95	134	92
CR	7.42	12.28	5.62	1.63	3.45	1.65	6.26	13.93	2.67	1.99	3.72	1.19	84	113	48	122	108	72
FA	57.84	89.50	69.50	72.77	84.11	89.31	46.32	111.50	72.54	71.00	114.81	52.97	80	125	104	98	136	59

Note: 0, 30, 60 are the ALA concentrations (mg/L).

## Data Availability

All the data is available within the manuscript.

## Abbreviations

ALA: 5-aminolevulinic acid; PH: Plant height; SD: Stem diameter; CB: Crown breadth; TNB: The total number of branches; Pn: Net photosynthetic rate; Tr: The transpiration rate; Ci: The intercellular CO_2_ concentration; Gs: Stomatal conductance; Fv/Fm: The maximum photochemical efficiency of photosystem II; qP: The photochemical quenching; ΦPSII: The actual photochemical efficiency of photosystem II; NPQ: The non-photochemical quenching; ETR: The electron transport rate; SOD: Superoxide dismutase; POD: Peroxidase; CAT: Catalase; MDA: Malondialdehyde; Chl: The total chlorophyll; Chl a: chlorophyll a; Chl b: chlorophyll b; WSC: water-soluble carbohydrate; PC: Polysaccharide; CR: carotenoids; FA: Flavonoids; PCA: Principal component analysis.
